# A small-molecule approach towards the Fountain of Youth: chemically induced pluripotent stem cells

**DOI:** 10.1093/nsr/nwac181

**Published:** 2022-08-27

**Authors:** Tuoping Luo

**Affiliations:** College of Chemistry and Molecular Engineering, Peking University

## Abstract

Generation and regeneration as an answer to disease treatment has been around for some time. Yet never have we come so close to reaching such ‘life-altering’ capabilities. Today, the field of regenerative medicine research focuses on replacing non-functional or dead cells with healthy ones, in order to repair or regenerate tissues and organs to restore normal functions. Pluripotent stem cells have the ability of long-term self-renewal and possess the potential to differentiate to all kinds of functional cells in humans. Therefore, how to directly obtain a large number of pluripotent stem cells from patients *in vitro*, to be grown into differentiated specific tissues and organs, has become one of the most important topics.

Six decades ago, Gurdon's group discovered that cell differentiation is a reversible process [1], laying down the foundation for cell reprogramming research. Commonly there are biological and chemical methods for the acquisition of pluripotent stem cells *in vitro*, which also aim to produce further differentiated specific tissues and organs. Fifteen years ago, Yamanaka's group first reported the acquisition of induced pluripotent stem cells (iPSCs) via overexpression of four transcription factors OSKM to the somatic cells [2]. Chemical reprogramming—using cell permeable small molecules to manipulate the cell fates—has also progressed significantly. Hongkui Deng at Peking University and his co-workers reported that a combination of small molecule compounds could induce pluripotent stem cells from mouse somatic cells with an induction efficiency as high as 0.2% in 2013 [3]. After long-term persistence and unremitting efforts, Deng's group announced the acquisition of chemically induced pluripotent stem cells (CiPSCs) from human fibroblasts through a step-wised chemical reprogramming strategy in 2022. This technology for preparing human CiPSCs solves the underlying technical bottleneck for the development of stem cells and regenerative medicine, and advances the application of cell reprogramming towards a new stage [4].

As the progress in human cell reprogramming led to sufficient resources of CiPSCs, chemically induced cell fate trans differentiation research also brought us surprises. Deng and colleagues not only demonstrated that small molecules can reprogram astrocytes into neurons in the adult mouse brain, which provides a potential approach for developing neuronal replacement therapies [5], but also constructed a bio-artificial liver device through directed differentiation of human pluripotent stem cells to hepatic cells [6]. Recently, Deng and colleagues established an efficient method for producing islet cells from human CiPSCs and demonstrated that these cells were able to ameliorate diabetes in non-human primates [7]. CiPSCs might be considered to have potential in the fields of cell therapy, drug screening and disease modeling, and are the most critical ‘seed cells’ in the field of regenerative medicine. Emerging as important regulators of cell fate, natural product small molecules and their derivatives have played an important role in Deng's work.

NSR spoke to Hongkui Deng about the highlights and possibilities of the field.

***NSR:*** It seems you worked on the areas of virology and immunology in your graduate and postdoc trainings. Why did you quickly shift your research direction to regenerative medicine when you started to build your independent group at Peking University?

***Deng:*** When I finished my postdoc training, I witnessed two major breakthroughs in the field of stem cell and regeneration. One was the birth of Dolly, the sheep in 1997—the first cloned mammal using a stem cell technique called somatic cell nuclear transfer, substantiating the potential of cellular reprogramming from a mature and developed state back to a primitive developmental state that could reproduce a live animal. This exciting work indicated that there might also be a chance that human cells could be manipulated by cellular reprogramming in the future, which should advance our understanding of human cell regulations and potentially bring about

**Figure 1:**
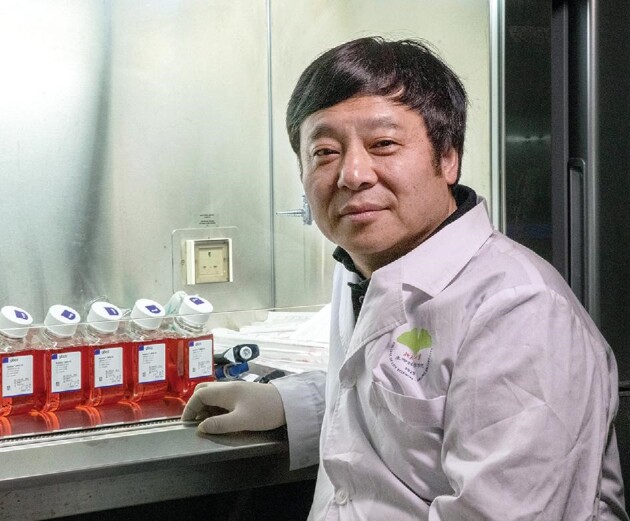
Hongkui Deng of the School of Life Sciences at Peking University, China *(courtesy of Hongkui Deng).*

Chemical reprogramming with small molecules, which are non-integrative to the genome and highly controllable, can regulate the cell fate with more flexibility and fine-tuning, without genetic manipulation.

—Hongkui Deng

the development of new cell-based treatments. The other breakthrough was the establishment of human embryonic stem cell lines in 1998. As we know from mouse embryonic stem cells (ESCs), ESCs are pluripotent and can become any cell type found in the body. In my view back then, it was truly a fascinating question how we could turn human ESCs into different types of functional cell types, and if this worked, it would bring a revolution to the methodology of how we approach human biology and how we develop cell-based therapies. These two groundbreaking discoveries represented the emergence of a new era for the development of regenerative medicine. After I established my group at Peking University, we started to explore how to culture human pluripotent stem cells, and then we were one of the first groups to convert these stem cells into pancreatic and hepatic lineages, which are now being adapted for medical treatments and tested in large animal models. Additionally, we were interested in developing new reprograming strategies, and for the first time showed that small molecules could reprogram mature cells into pluripotent stem cells using no biological materials. This strategy is now broadly known as chemical reprogramming and is being further explored by different research groups.

***NSR*:** The discovery of the CCR5 receptor as the major co-receptor for HIV in your postdoc research laid the foundation for the development of small-molecule medicines to inhibit HIV-1 entry (for instance, Maraviroc). Your group has recently reported the first case of CCR5-edited stem cell therapy on an HIV patient together with collaborators. What do you think of the potential of iPSCs for AIDS treatment?

***Deng:*** Curing AIDS, which is caused by HIV infection in humans, is a great long-term challenge. In 1996, with colleagues working in Dan Littman's lab, I found CCR5 to be the major co-receptor used by HIV for the infection of human cells. Following this discovery, CCR5 became a target for the development of anti-HIV treatments. One of the best examples is the ‘Berlin Patient’, in whom transplantation of hematopoietic stem cells (HSCs) with a naturally occurring CCR5 mutation prevented HIV-1 infection in immune cells, resulting in non-detectable HIV in his body. Consequently, we thought that we could use gene editing to create CCR5-deleted HSCs for treating HIV infection. After numerous attempts using animal models, we developed a clinical trial to use HLA-matched CCR5-deleted HSCs to treat a patient who was diagnosed with both leukemia and HIV infection. We found that the modified HSCs carrying the ablated CCR5 persisted for at least 19 months after transplantation, supporting the long-term engraftment and safety of clustered regularly interspaced short palindromic repeat (CRISPR)-edited HSCs, which gives us confidence that this strategy is promising for treating HIV infection in the future. However, along with other clinical trials exploring HSC/gene-editing-based therapy, the editing efficiency in HSCs is still very low, resulting in a low frequency of desired functional immune or blood cells. This issue could be overcome by using iPSCs, which can now be gene edited more easily. For example, we could generate CCR5-deleted iPSC lines and convert these cells to HSCs for transplantation use. This is definitely an exciting and promising area; nevertheless, combined iPSC and gene-editing treatments are still under development and should not be used on patients before we have a clear understanding of their safety and efficacy.

## REGENERATIVE MEDICINE

***NSR*:** What are the key areas of progress that have shaped the regenerative medicine field within the past two decades?

***Deng:*** In my view, there have been several major areas of progress that have greatly promoted the development of regenerative medicine within the past two decades. First, in the research into reprogramming, two major reprogramming techniques have been invented in addition to the previous one of somatic cell nuclear transfer established in the 1950s. In 2006, Shiya Yamanaka developed a transcription-factor-based reprogramming strategy that boosted research on cell fate conversion, including the generation of pluripotent stem cells from somatic cells and trans differentiation across developmental lineages. In 2013, our group developed a chemical reprogramming strategy in which cell fate manipulation could be achieved by using just chemicals without the involvement of biological materials such as oocytes or transcription factors. These new technologies represent advances in basic methodology for studying cell biology, laying down a foundation to develop cell-based regeneration therapies. Second, the differentiation of pluripotent stem cells has shown great power in disease modeling, drug discovery and cell therapies in the past 10 years. With differentiation, we have been able to generate a large number of functional cell types, with a high purity, for developing cell replacement therapies, and to construct complex organoid systems to study human development and diseases in a dish. Another aspect, in my opinion, is the combination of gene-editing tools in stem cell research. By using gene-editing techniques, we can edit stem cells and customize a function or a phenotype of interest. As mentioned above, our proof-of-concept study of CCR5-modified HSC transplantation is an example of how this combination works. Indeed, such a combination amplifies our opportunities to study cell behaviors and to develop new cell-based treatments.

***NSR*:** What are the remaining problems that are yet to be addressed, in your opinion?

Chemical reactions are the essence of almost all biological events, including development and reprogramming.

—Hongkui Deng

***Deng:*** With respect to clinical application, the most important elements in my mind are functionality and safety, which are thought to be major challenges for stem cell transplantation research. It is still not very easy to obtain functionally mature cells from pluripotent stem cells that are similar to their counterparts found in the body. This is the foundation for the application of cells derived from iPSCs. In addition to functionality, the other element is the elimination of safety concerns caused by the

genomic integrity and tumorigenic potential of human pluripotent stem cells. Recently, a large-scale and whole-genome analysis of human induced pluripotent stem cells (hiPSC) derived from the transcriptional-factor-based approach has shown that a high percentage of hiPSC lines possess cancer-driver-gene-related mutations. Chemical reprogramming with small molecules, which are non-integrative to the genome and highly controllable, can regulate the cell fate with more flexibility and fine-tuning, without genetic manipulation. Therefore, it is possible that we will obtain the safest pluripotent stem cells and other cell lineages via the small-molecule approach, which could tremendously promote the development of regenerative medicine. Moreover, using engineering technologies, we devised a bio-artificial system that might help patients via external devices. Instead of putting cells into the patient’s body, we used stem-cell-derived hepatocytes to create a dialysis-like device that treats liver diseases by clearing blood toxins. Such a bio-artificial liver machine may ease the symptoms of liver failure and prolong the time until a liver transplant is needed. This external device, physically separating iPSC derivatives from the patient, could mitigate certain safety issues as the cells can easily be removed if issues arise.

***NSR*:** What are the key issues that need to be overcome in the future clinical application of pluripotent stem cells, in particular CiPSCs?

***Deng:*** In addition to the challenges of obtaining functionally differentiated cells and addressing safety concerns, it is also important to develop a platform for generating final cell products on a large scale. Furthermore, it will be more attractive and economical to prepare off-the-shelf cell products at the good manufacturing practice (GMP) level for future clinical application of pluripotent stem cells. CiPSCs have intrinsic advantages in cell manufacturing and the current issue is how we translate these advantages into practice. For example, unlike other systems, the process of generating CiPSCs does not require researchers with extensive experience of reprogramming or the detection of exogenous genetic elements, which facilitates clinical application. To this end, we will also need to optimize the reprogramming system by establishing a more efficient and robust method to facilitate the clinical-grade CiPSC lines. This optimization work requires us to further investigate the mechanisms underlying human chemical reprogramming, which would provide new targets for the invention of robust chemical reprogramming systems.

## THE SMALL-MOLECULE APPROACH

***NSR*:** As a biologist, you paid particular attention to, and persisted in, studying the regulation of cell fate using chemicals instead of biological molecules such as transcriptional factors and cytokines. Why?

***Deng:*** Chemical reactions are the essence of almost all biological events, including development and reprogramming. In the body, cells receive external signals from the environment and are directed to change their cell fate, identity and functionality. In addition, in lower animals, external stimulation sufficiently triggers cell dedifferentiation for regeneration after injury. All of these indicate that external stimulation represents a more natural mechanism for cellular reprogramming. However, external-stimulation-induced cellular reprogramming in human cells is challenging, prohibiting a restart of cell plasticity and cell fate regulation. This is because of a stable epigenetic landscape in human cells, arising from evolution, which reduces cell plasticity to protect a committed and specialized cell identity. Once we overcome these hurdles, external-based cell reprogramming may permit efficient cell fate manipulation in human cells, which will lay a foundation for developing highly controllable and flexible therapeutic reprogramming strategies. Chemical reprogramming using small molecules to perform external stimulations provides a promising approach to manipulating cell fate. There are many advantages to using small molecules as they are non-integrative to the genome, highly controllable and easy to optimize, standardize and manufacture. More importantly, for effective cell fate manipulation, synergistically regulating multiple signaling and targets are required. The traditional transcriptional-factor-based reprogramming approach is limited in terms of the flexibility of combinations and duration. In comparison, the combinations of small molecule can be simply implemented to effectively manipulate cell fates in a temporally and spatially flexible manner. Therefore, the chemical reprogramming approach could be ideal for developing therapeutic reprogramming strategies in a highly controllable and easy-to-standardize manner.

***NSR*:** What has been the most exciting moment during the CiPSC research?

***Deng:*** In 2013, our proof-of-principle study demonstrated that mouse somatic cells can be induced to a pluripotent state using only small-molecule treatment. When we first completely confirmed our mouse-cell-reprogramming results, it was an amazing moment that opened a new chapter of how we think about reprogramming. For the first time we could reprogram cells mainly without the use of key biological materials, such as oocytes and transcription factors, which represents a new pathway of cellular reprogramming. Such a simple exposure to a chemical cocktail with four small molecules was able to generate pluripotent stem cells from developed mature cells. At that moment, we thought we would have unprecedented opportunities to manipulate cells for basic research and for the development of new cell-based interventions. Another very impressive finding is the identification of the regeneration gene program activated in human cells during chemical reprogramming. In lower animals such as salamanders, cells possess a powerful regenerative potential for the activation of regeneration-related genes after tissue injury. In contrast, owing to evolution, the regeneration potential in humans is highly restricted by their specialized cell identities and functionalities. Interestingly, during the conversion of human somatic cells to human chemically induced pluripotent stem cells (hCiPSC), we identified a population of human cells that are similar to blastema cells, a progenitor cell type that is transiently formed for limb regeneration in salamanders. These results highlight that chemical reprogramming not only provides a new platform for the generation of pluripotent stem cells, but also holds great promise with regard to regulating human regeneration. As paradigms for translating small molecules into medicines have been established, this chemical approach paves the way for the development of simple pharmacological strategies for human regeneration.

***NSR*:** It took quite some time (nine years) from your report on the first CiPSCs in mice to the successful reprogramming of human somatic cells. What were the key issues encountered along the way and how did your team overcome them?

***Deng:*** For a long time, it had been a huge challenge for chemical reprogramming to induce pluripotent stem cells from human somatic cells. We and colleagues from other laboratories working on chemical reprogramming reason that human somatic cells have a stable epigenome with reduced plasticity, as a result of evolution, and hence could be refractory to chemical stimulations that drive epigenome-wide reprogramming. To look for a solution, we deeply considered many natural reprogramming systems, and an important clue we found to overcome this issue was provided by the remarkable dedifferentiation processes in certain lower animals. In animals like axolotls, the injured somatic cells can respond to external signals to initiate cell dedifferentiation and generate a plastic state. Such a plastic state has relatively open chromatin architecture with increased accessibility, which is crucial for inducing a new cell fate. Based on this hypothesis, we successfully induced an intermediate plastic state with a regenerative gene program at the early stage of reprogramming, which is a key step for the subsequent induction of hCiPSC cells.

## THE ROLE OF NATURAL PRODUCTS

***NSR*:** Given that there are different sources of small molecules, how did you find and choose the small-molecule compounds during the reprogramming process?

***Deng:*** For chemical reprogramming, phenotypic- and functional-based screening is a powerful strategy to identify small molecules that regulate a specific biological process. Even though high-throughput screening is an important method for effective identification of powerful compounds, we did not screen anything without a focus. Following each screening experiment, we often performed mechanistic investigations, the results of which would direct us towards a strategy for the next round of chemical screening. For example, we always check key cell-signaling pathways, cell proliferation and death pathways, and epigenetic modifiers, which, taken in conjunction with previous literature, provide us with clues to choose an appropriate library for subsequent investigation. In addition, as chemicals often regulate multiple targets during reprogramming, we also investigate the roles of individual small molecules via decomposition, followed by recompositing small-molecule combinations, which is an important strategy to search for small-molecule compounds promoting different reprogramming stages.

***NSR*:** What is the role of natural products—compounds originated from various natural sources, such as plants, bacteria, fungi, insects and higher-order animals—in facilitating the generation of CiPSCs or other reprogramming processes?

***Deng:*** During the reprogramming process, we found that compounds from natural sources play an important role. For example, Oct4 is the core transcription factor for the pluripotent gene network, the activation of which is the most important step for the induction of CiPSCs. However, it is very challenging to activate the expression of endogenous Oct4, as it is strictly restricted in somatic cells. We identified that forskolin, a labdane diterpenoid produced by the plant *Coleus barbatus* and an agonist of adenylate cyclase, is essential for activating the endogenous Oct4. In addition, forskolin alone is able to replace exogenous Oct4 and c-MYC to induce the generation of iPSCs under the combination of KLF4 and SOX2. This natural product is one of the most potent small molecules we identified for cell reprogramming.

***NSR*:** What is the uniqueness of natural products in comparison to drug-like molecules invented by pharmaceutical companies?

***Deng*:** Natural products are formed during a long-term process of evolution and selection, which is one of their most important characteristics compared with synthetic compounds. Given this uniqueness, natural products possess an unparalleled diversity of structures, enabling them to cover a broader spectrum of biologically relevant chemical space than synthetic compounds.

Natural products are formed during a long-term process of evolution and selection, which is one of their most important characteristics compared with synthetic compounds.

—Hongkui Deng

In addition, natural products bear a higher degree of stereochemical complexity, which boosts their potential to modulate difficult targets. It has been reported that >30% of U.S. Food and Drug Administration (FDA)-approved small-molecule medicines are natural products or their derivatives. Moreover, natural products also have the ability to be substrates for transporter systems, which are important for manifesting their biological activity by accessing the intracellular site of action.

## LOOKING INTO THE FUTURE

***NSR*:** What kind of small molecules are desired but have yet to be reported for taking CiPSCs forward?

***Deng:*** Through mechanisms analysis, we can identify many targets that are important to regulate the process of cellular reprogramming, such as transcription factors or enzymes that control epigenetic events. However, potent small molecules to efficiently regulate these targets are still lacking. Recently, the development of artificial intelligence systems such as the AlphaFold, which could predict the 3D structures of many proteins from amino acid sequences, provides unique opportunities, via the utilization of protein structure information, for the screening and identification of desired small-molecule compounds.

***NSR*:** Is there any other field that will attract your particular attention in the next decade?

***Deng:*** One attractive field to me is aging research, which is important for understanding aging-related issues and for developing new treatments for many diseases. Recently, we and collaborators developed a prodrug strategy to selectively eliminate senescent cells based on their increased activity of lysosomal β-galactosidase, which provides a novel strategy to develop anti-aging interventions. In addition, I am also interested in research into cell totipotency and the derivation of totipotent stem cells *in vitro*. This is the key for us to understand a fundamental biological question—the origin of life. If we could artificially recapitulate totipotent stem cells *in vitro*, it may provide not only a good model for embryo development but also new approaches for animal cloning. In addition, totipotent stem cells would be ideal for whole-organism genome editing and for generating desired cell lineages. Recently, we have reported the possibility of deriving totipotent-like stem cells that can be stably maintained *in vitro*. This study opens a new path towards fully capturing totipotent stem cells *in vitro*.

***NSR:*** What are your advices to young researchers?

***Deng:*** I would recommend that young researchers focus on the most important questions in their fields. If I was a younger researcher, I would have told myself to stay positive, persistent and passionate. Working on a hard scientific question helps to keep research passions and motivations alive, but this process is also challenging. To address the fundamental questions in science, persistence and courage are most important for overcoming a series of potential challenges. I also suggest young researchers prepare themselves for multidisciplinary research and collaborations by building a broad knowledge base. Networking is an important part of research, where you meet experts in other fields who are potentially important to you in terms of developing collaborations. Networking also offers the opportunity to exchange techniques, research methodology, scientific problems and different ways to approach and solve problems. I would like to encourage young researchers to develop a habit of networking so that they can always seek advice from experts representing a broader community of research.

## Notes

Tuoping Luo is a principal investigator at the College of Chemistry and Molecular Engineering, Peking University.
